# Separation of Epiphyses

**Published:** 1901-09-14

**Authors:** 


### SEPARATION OF EPIPHYSES.

When as the result of injury the continuity of a bone is destroyed it is not unnatural to speak of the lesion as a fracture. Often, however, what has happened is but the separation of the epiphyses, and it is well to bear in mind that this is a lesion which is by no means confined to infancy. Mr. John Poland[1] says that although it was formerly thought that infancy was the usual time for these injuries to take place, later experience has shown that the most common period for separation to occur is between 11 and 18 years of age. There is, he says, an enormous preponderance in number of separations in the male over the female sex; and the epiphyses of the upper are more often involved than those of the lower extremity. Practical experience has shown that the various epiphyses of the lower end of the humerus taken collectively are most frequently separated. Of the single epiphyses the order is as follows: upper epiphysis of humerus, lower epiphysis of radius, lower epiphysis of femur, lower epiphysis of tibia. Taken in accordance with the number of pathological specimens in museums the order is different, but that is doubtless due to the fact that it is only lesions which are fatal that arrive on the shelves of museums. It may be taken as an almost invariable rule for the separation to pass through the juxta-epiphysial region or the ossifying layer adjoining the conjugal disc of cartilage, so that the latter still remains with the epiphysis. This blue layer of cartilage is very evident in compound separations. Sometimes the separation is complete and pure, extending through the whole length of the epiphysial line, sometimes it is partial or incomplete, proceeding through a certain portion of the line and finishing its course as a fracture through the diaphysis of the bone. The latter is the more common form, so that in many cases the lesion here discussed may have to be considered as a complication of a fracture rather than as an independent injury. An extremely important matter to remember is that separation of an epiphysis may occur without displacement, and yet may be followed by the serious after-consequences which sometimes result from this injury. Such cases are very liable to be overlooked, to receive no treatment, and consequently to be followed by arrest of growth, periostitis, suppuration, etc. The signs are few. While there is the history of an injury in a young subject with absence of deformity about the joint, there are present some swelling and pain about the epiphysial region, mobility of the epiphysis at the same point, and occasionally some soft crepitus or ecchymosis. Unfortunately the X-rays do not assist us in the diagnosis, the appearance of the shadow picture being precisely similar to that of the normal end of the bone. Again, without actual separation there may be sprains. These may cause crushing of the spongy tissue of the diaphysis with bending, compression, or fracture of the compact tissue. The symptoms are few—loss of power of the limb, with swelling about the juxta-epiphysial region, while the neighbouring joint remains free. Finally there may be incomplete separation in infants. This lesion, like the last-mentioned one, favours the development of bacteria and may lead to tuberculous disease. It is to be noted that although an epiphysis may not be displaced at first it may gradually become so later, this being commonly the case where the injury has been regarded as a simple sprain, and the patient has been allowed to use the limb. As to symptoms, when the displacement is marked, the injury exhibits more resemblance externally to a dislocation than to a fracture, and such an accident to the head of the humerus has often led to the upper diaphysal end of the bone being mistaken for a displaced head, and to attempts at reduction being made. Abnormal mobility at the epiphysial line is the chief sign of all. Muffled crepitus is characteristic when present, but it is often absent, from complete displacement or from locking of the fragments, or from the interposition of periosteum or muscle. The X-rays are often of use in diagnosis, but a skiagram of an uninjured joint of a person of the same age, and taken under precisely the same conditions is always necessary for the interpretation of the skiagram of an injured epiphysis. The rays must be regarded as supplementing, not as taking the place of other means of investigation, but they are of especial value in cases in which the swelling of the soft parts causes difficulty in ascertaining the condition within. In considering the consequences of separation of an epiphysis we must bear in mind one of its most frequent complications, namely, stripping of the periosteum from the diaphysis. Among these consequences are necrosis of the diaphysis, exuberant development of callus interfering with the movement of the joint, deformity from incomplete reduction, paralysis and gangrene, and in some cases atrophy of muscles, from injury to nerves and vessels, and arrest of growth of the bone which sometimes occurs as the direct result of inflammation and destruction of the cartilage cells of the conjugal disc. Where parallel bones exist, as in the forearm and leg, the uninjured bone may continue its normal growth, and thus deviations of the hand or foot may be produced. In regard to treatment the replacement must be immediate and exact. The manipulative procedures to effect this are often more allied to those required in the case of dislocations than of fractures, and an accurate knowledge of the contiguous surfaces of the epiphysis and diaphysis is of the greatest moment in the reduction of the displacement.

[1] Practitioner, Sept.

